# Zika virus infection and acute transverse myelitis: a comprehensive systematic review

**DOI:** 10.1590/S1678-9946202466066

**Published:** 2024-12-06

**Authors:** Bianca Aparecida Colognese, Nayara Argollo

**Affiliations:** 1 Universidade Federal da Bahia, Hospital Universitário Professor Edgard Santos, Salvador, Bahia, Brazil

**Keywords:** Transverse myelitis, Zika virus, Spinal cord diseases

## Abstract

The Zika virus (ZIKV) has been associated with several complications, including acute transverse myelitis (ATM), an acute inflammation of the spinal cord, with rapid development of motor, sensory and dysautonomic symptoms. It is a rare disease, and its clinical features, as well as differences in relation to idiopathic ATMs, are still not completely known. The objective of this paper is to review the literature in search of clinical features and complementary exams of ATM post-ZIKV infection, alone or in association with other neurological conditions (mixed diseases), as well as its treatments and prognoses. The search was made on 5 databases, using the PRISMA methodology (Preferred Reporting Items for Systematic Reviews and Meta-Analyses). Nine articles were selected (total of 20 subjects), which were divided between isolated ATM and mixed neurological syndromes with ATM. The study found a predominance of individuals aged 20 to 30. Among the six subjects in the mixed group, three were over 50 years old. The median prodromal period was 2 days for the mixed diseases group and 7 days for the isolated ATM group. Some individuals in the isolated ATM group exhibited signs of dysautonomia, such as syncope, postural lability, and arrhythmia. The mixed group had a higher incidence of coinfections, with 4 cases compared to 1 case in the isolated ATM group. Over 50% of the individuals had moderate to moderately severe disability. These findings suggest that severe conditions may progress to significant sequelae, highlighting the need for prompt diagnosis and treatment, particularly during endemic periods.

## INTRODUCTION

The Zika Virus (ZIKV), first discovered in 1947 in Uganda, in the Zika forest, has gained attention in the past years, after an epidemic started in October 2015 in Recife, which later spread to Brazil and part of the world. The infectious agent is transmitted mainly through the *Aedes aegypti* mosquito, which is also a vector of other flaviviruses, such as dengue, yellow fever and chikungunya. Other transmission routes are sexual, blood and vertical. The disease manifests with a diffuse and pruritic maculopapular rash, low-grade fever (37.8 to 38.5 °C), arthralgia, conjunctivitis, retro-orbital pain, headache, myalgia and asthenia^1-6^.

Several complications are associated with ZIKV infection, including Guillain-Barré syndrome, congenital ZIKV syndrome, meningitis, myelitis, and mixed syndromes such as meningoencephalitis, encephalomyelitis, meningomyelitis, and myeloradiculitis^2-5,7^.

Acute transverse myelitis (ATM) is a segmental inflammation of the spinal cord that can be acute or subacute and is rare in the world population^8^. This condition leads to the development of motor and sensory symptoms, and to dysautonomia, which can manifest with syncope, postural lability, arrhythmia, urinary incontinence or retention, fecal incontinence, constipation, or sexual dysfunction. Motor symptoms usually retain a spinal cord level, with rapidly progressive paraparesis, initially flaccid (spinal shock) and later spastic. Sensory symptoms include paresthesia, dysesthesia and pain^7-9^. Magnetic resonance imaging (MRI) shows an alteration in the spinal cord signal, with gadolinium enhancement. In the cerebrospinal fluid (CSF) there may be pleocytosis^10-12^. It is estimated that 30%-60% of idiopathic cases are related to previous infection, which causes neurological damage by direct infection to cells or by the action of inflammatory mediators^7,8,10-13^. Regarding ZIKV, the pathophysiological mechanism is not completely understood. Antigenic mimicry with autoantibody development, similar to multiple sclerosis and neuromyelitis optica, may be the causative mechanism^13^.

The occurrence of ATM after ZIKV infection is rare, and its clinical picture and differences from idiopathic ATMs are not fully elucidated. This study aims to review the literature comprehensively and systematically to search for clinical characteristics, treatment and evolution of ATM after ZIKV infection, whether it occurs alone or in association with other neurological conditions.

## MATERIALS AND METHODS

A systematic literature review was carried out using the PRISMA methodology^14^, in the following databases: MEDLINE (Medical Subjects Online), SciELO (Scientific Electronic Library Online), LILACS (Latin American and Caribbean Literature in Health Sciences), CAPES (Coordination for the Improvement of Higher Education Personnel), and Elsevier Scopus.

**Figure 1:**
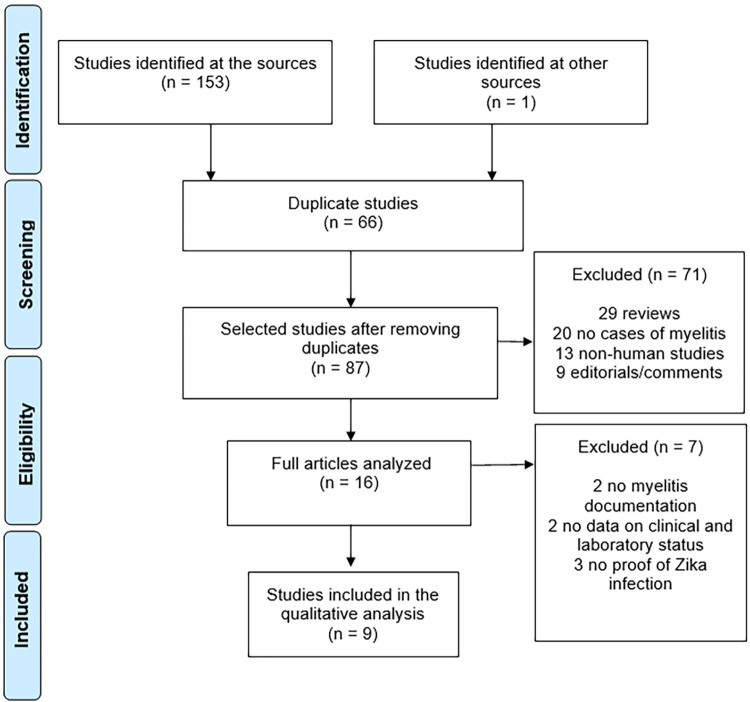
PRISMA flow diagram.

The searches were conducted between May 13, 2019, and November 18, 2023. A total of 154 studies were initially identified, of which 66 were duplicates. During the screening phase, 71 articles were excluded because they were reviews, commentaries, editorials, involved non-human subjects, or did not present cases of myelitis. Sixteen full articles were analyzed, of which 7 were excluded according to the exclusion criteria. The process was detailed in the flow diagram (Figure 1).

To search the databases, two groups of descriptors were established. The first group related to ZIKV, and the second to ATM. In the latter, keywords related to “diseases of the central nervous system” were included in order to reach a greater number of studies.

Among the words of the same group, the Boolean operator “OR” was used, and between the groups, “AND”, in the platforms that allowed this feature. For the others, at least 3 words were selected from the first group, and 5 from the second, and the searches were carried out one-by-one.

First group of descriptors:

Zika; ZikV; infection, Zika virus; ZikV infection; Zika virus disease; Zika fever.

Second group of descriptors:

Myelitis; spinal cord inflammation; inflammatory myelopathy; transverse myelopathy syndrome; transverse myelitis; acute transverse myelitis; subacute transverse myelitis; postinfectious myelitis; nervous system diseases; neurologic disorders; mielite; doença desmielinizante

The gray literature was searched in the references of articles, conference proceedings and the CAPES theses and dissertations database.

Inclusion criteria: case reports, case series, case-control, cross-sectional and cohort studies. No language restriction or publication date limitation were adopted.

Exclusion criteria: unavailability of the full text after contacting the author, no report of exams proving ZIKV infection, absence of data on the clinical and laboratory condition of the mentioned patients, articles that did not meet the inclusion criteria, studies in non-humans, repeated works or with the same database, literature reviews, editorials and commentary.

For this study, cases of ZIKV infection were defined as those with positive serology or RT-PCR for Zika with a compatible clinical history. Cases of ATM were defined as those that presented a compatible clinical picture and imaging tests, or whose authors described the criteria used for the diagnosis.

Data were collected on sex, age, diagnostic methodologies, prodrome time, clinical status, complementary exams, evidence of coinfection, autoantibodies, immunity against arboviruses, treatment, outcome, time of discharge, non-neurological complications and sequelae. The modified Rankin Scale (mRS)^15^, was applied to assess the degree of sequelae of the individuals, when it had not been applied by the original authors (Table 1). Data were divided between isolated MTAs and mixed conditions (encephalomyelitis, meningomyelitis and myeloradiculitis).

**Table 1:** Modified Rankin scale for post-stroke functional assessment15.

Degree	Description	
0	No symptoms	
1	No significant deficiencies, despite symptoms	Able to conduct all usual duties and activities
**2**	**Slight disability**	Unable to carry out all the activities as before, but it is able to look after own interests without assistance
**3**	**Moderate disability**	Requires some help but able to walk unassisted (may use cane or walker)
**4**	**Moderately severe disability**	Unable to walk unassisted and unable to attend own physiological needs without assistance
**5**	**Severe disability**	Confined to bed, incontinent, requiring constant nursing care and attention
**6**	**Death**	

## RESULTS

Nine articles were selected, totaling 20 subjects. All publications occurred between 2016 and 2019, with 3 in 2017 and 4 in 2018. Six were case reports; and one of each: case series, retrospective case-control and cohort (Table 2). Five articles were from Brazil, 3 from Colombia and 1 from the French Republic. Among the 20 subjects, 14 (8 studies – 70%) had isolated ATM and 6 (3 studies – 30%) had a mixed condition (Table 3).

**Table 2:** Selected articles, clinical pictures, diagnostics, treatments and sequelae of the isolated ATM group.

Article	Study design	Sample size, sex and age	Clinical picture	Diagnosis, coinfection, previous immunity against other arboviruses	Treatment, evolution, days of hospitalization	Sequelae/Severity according to the mRS
1. Anaya *et al.* ^16^	Case control retrospective	2 F and 4 M (average: 22.5 years; IQR: 17.5 - 32)	Average 32 days of prodrome (IQR: 13 – 96). Symmetric paresis (4), paraparesis (5), Paresthesia (4), trunk weakness (2), hyperreflexia (6), sensory level (6), autonomic signs (3), urinary retention (3), arrhythmia (2), pressure lability (1), paralytic ileus (3)	Positive IgG (6). CSF: pleocytosis (2). Not tested in 3 cases. MRI: Change in intramedullary signal with contrast enhancement (4). Involvement of 3 vertebrae (3).* Immunity against other arboviruses: IgG+ for dengue in serum (6), IgG+ for chikungunya (1)	Corticosteroid therapy (5). Discharge within 6 days (5)	ND
2. Casagrande *et al.* ^17^	Case report	1 M (23 years old)	40 days of prodrome. Paraplegia. Muscle spasms. Paresthesia. Backache. Hypoesthesia w/ level at T4. Hyperesthesia in thorax and ULs. Urinary retention, constipation and dysphagia. Bilaterally indifferent plantar cutaneous.	Positive IgM. CSF: increase in cells and proteins. MRI: Intramedullary hypersignal in the cervical spine on T2-weighted contrast enhancement.	Corticosteroid therapy with 0.5 mg/kg/day prednisone. Strength improvement in ULs. Dev. of allodynia in ULs. No improvement of the rest of the picture. Grade IV decubitus sacral ulcer.	LLs paraplegia, urinary incontinence and evacuation through enema in return visit (mRS 5)
3. Lima *et al.* ^18^	Case report	1 F (24 years old)	3 days of prodrome. Paraparesis. Unilateral headache. Syncope. Tonic-clonic seizure.	Positive PCR. MRI: Changes compatible with transverse myelitis. Diagnosis of SLE 15 years ago (in remission)	IVIG 2 g/kg associated with methylprednisolone 125mg 1x/day for 5 days, followed by pulse therapy with methylprednisolone 1 g for 5 days	ND
4. Mécharles *at al.* ^19^	Case report	1 F (15 years old)	7 days of prodrome. Left-sided hemiparesis, paresthesia and hypoesthesia. Low back pain and in left LL and UL. Urinary retention. Hoffman +. Glasgow 15.	Positive PCR. CSF: no changes. MRI: Intramedullary hypersignal in the cervical and thoracic spine on T2.	Corticosteroid therapy with 1 g/day methylprednisolone for 5 days; improvement;	Moderate weakness in LLs, deamb. without help in 1 month. (mRS 3)
5. Mehta *et al.* ^20^	Case report	1 F (59 years old)	7 days of prodrome. Spastic quadriparesis. Paresthesia. Hypoesthesia. Gasglow 15.	Positive IgM; CSF: increase in leukocytes and protein; MRI: hypersignal at cervical and thoracic levels; AB: antigangliosides GM1, GD1a, 1b; Coinfection with dengue and chikungunya.	IVIG and 2 courses of corticosteroid therapy; improvement with corticosteroid therapy. Pulmonary edema after IVIG.	Moderate. Deamb. without help in the 4th month. (mRS 3)
6. Neri *et al.* ^21^	Case report	1 F (38 years old)	9 days of prodrome. Flaccid paraplegia. Urinary retention. Intestinal constipation. Presence of pyramidal signs. Hypoesthesia.	Positive IgM and PCR. CSF: no changes. MRI: Intramedullary hypersignal in cervical and thoracic spine on T2 with contrast. AB: positive anti-MOG.	2 courses of corticotherapy with methylprednisolone 1g/day for 5 days. Improvement with corticosteroids.	No motor deficits. Deamb. without help at hospital discharge. Hypoesthesia and muscle spasms in mmii. (mRS 1)
7. Palacios *et al.* ^22^	Case report	1 M (23 years old)	15 days of prodrome. Flaccid paraplegia. Abdominal paresthesia. Hypoesthesia. Sensory level at T7. Pelvic pain. Urinary retention. Hyperreflexia.	Positive PCR. CSF: Pleocytosis and low protein levels. MRI: Intramedullary hypersignal from C1 to the conus medullaris on T2.	Corticosteroid therapy (methylprednisolone 1g/day for 5 days) and 5 plasmapheresis sessions; No improvement with corticosteroids. Improvement after plasmapheresis.	Strength 4/5, deamb. with the aid of a cane at hospital discharge. (mRS 3)
8. Silva *et al.* ^23^	Observational prospective cohort	1 M and 1 F (23 and 43 years old)	7 days prodrome (both). Paresis in LLs (both), back pain (1), pain in LLs (1) sensory deficit (both), ataxia (1).	Positiv e IgM (both). MRI: Intramedullary hypersignal on T2 (both).	Corticosteroids (both), plasmapheresis (1). Discharge at 10 and 11 days.	Light. Able to look after own interests without assistance at 3 months (both) (mRS 2)

F = female; M = male; IQR = interquartile range; ND = no data; IVIG = Intravenous immunoglobulins; CSF = cerebrospinal fluid; MRI = Magnetic Resonance Imaging; GBS = Guillain Barré syndrome. AB = antibodies. Y/ = without; C/ = with; N = nerves; UL = upper limb(s); LL = lower limb(s); In parentheses = number of cases. *ATM diagnosis based on Transverse Myelitis Consortium Working Group Criteria^15^.

**Table 3:** selected articles, clinical conditions, diagnoses, treatments and sequelae of the mixed condition group.

Article	Study design	Sample size, sex and age, condition	Clinical picture	Diagnosis, coinfection, previous immunity against other arboviruses	Treatment, evolution, days of hospitalization	Sequelae/Severity according to the mRS
1. Anaya *et al.* ^16^	Case control retrospective	1 F (25 years old). Myeloradiculopathy.	2 prodrome days. Paraparesis. Hyporeflexia. Abdominal weakness. Hypoesthesia. Urinary retention	Positive IgG. ND on complementary exams*. Immunity against other arboviruses: IgG + for dengue and chikungunya.	ND	ND
5. Mehta *et al.* ^20^	Case series	M (26 years old). Myeloencephalitis.	1 prodrome day. Hyperreflexive spastic quadriparesis. Paresthesia. Sensory level at T5. Hypoesthesia. Urinary retention. LMN syndrome in n. facial. Supranuclear gaze palsy. Glasgow 13.	IgM and PCR positive; CSF: leukocytosis. MRI: Intramedullary hypersignal. Abnormality in the cerebellar peduncles signal. Coinfection with dengue.	IVIG + Corticosteroids. Orotracheal intubation and subsequent improvement.	No significant impairment despite symptoms at 4 months. (mRS 1)
		F (80 years old). Myeloencephalitis + subclinical meningitis.	5 days of prodrome. Hyporeflexive flaccid quadraparesis. Headache. Confusion. Glasgow 14.	positive IgM. CSF: increase in leukocytes and protein. MRI: Hypersignal in cervical and thoracic columns. Hypersignal in temporal lobes, amygdala, periventricular, and pachymeningeal enhancement. Coinfection with chikungunya.	IVIG. No clinical improvement.	Unable to walk and attend to physiological needs without assistance at 2 months. (mRS 4)
		M (65 years old). Myeloradiculitis.	0 prodrome days. Reflexive flaccid paraparesis. Paresthesia. Hypoesthesia. Sensory level at T11. Urinary retention. Glasgow 15.	Positive IgM and PCR. CSF: leukocytosis; MRI: no changes.* ENM: acute motor and sensory axonal neuropathy. Coinfection with Chikungunya.	IVIG. Corticosteroid therapy. Without clinical improvement	No improvement at 3 months (mRS 4)
		F (56 years old). Myeloradiculitis.	4 days of prodrome. Reflexive flaccid quadriparesis. Paresthesia. Sensory level at C7. Hypoesthesia. Glasgow 15.	Positive PCR. CSF: increase in protein. MRI: no changes.* ENM: acute motor and sensory axonal neuropathy. Coinfection with Chikungunya	IVIG. Corticosteroid therapy. Without clinical improvement	No clinical improvement at 1 month. (mRS 5)
9. Mancera-Páez *et al.* ^24^	Case report	1 F (26 years old). GBS, transverse myelitis and encephalitis.	1 prodrome day. Paraparesis with foot drop. Epigastric pain radiating to the back. Urinary retention. Ascension of weakness after 2 days w/ dev. of flaccid quadriplegia, hypoxemia and confusion. Areflexia. Hypoesthesia. Medullary level at T6. Unilateral optic neuritis. Hoffman’s sign +. Indifferent plantar cutaneous	Positive PCR and IgG. MRI: Multiple hypersignals in cervical and thoracic spines without contrast enhancement. Periventricular hypersignal, subcortical areas and brainstem. EMG: LL denervation signals; Immunity against other arboviruses: diagnosis of dengue and chikungunya in 2014	IVIG and corticosteroid therapy (methylprednisolone). Improvement of the level of consciousness. Basal pneumonia and antibiotic-resistant *Acinetobacter baumannii* septicemia. Respiratory support. Discharge at 6 weeks.	Flaccid paraplegia with areflexia. Hypoesthesia in mmii. Start of ambulation with support after 1 year. Persistence of urinary incontinence and constipation. (mRS 4)

ND = no data; IVIG = Intravenous immunoglobulins; CSF = cerebrospinal fluid; GBS = Guillain Barré syndrome; MRI = Magnetic Resonance Imaging; EMG = electromyoneurography; AB = antibodies; LMN = Lower Motor Neuron; Y/ = without; C/ = with; N = nerves; Uls = upper limbs; LLs = lower limbs; In parentheses = number of cases. *ATM diagnosis based on Transverse Myelitis Consortium Working Group Criteria^15^.

The studies that brought cases of isolated ATM were: Anaya *et al.* ^16^, Casagrande *et al.* ^17^, Lima *et al.* ^18^, Mécharles *at al.* ^19^, Mehta *et al.* ^20^, Neri *et al.* ^21^, Palacios *et al.* ^22^, Silva *et al.* ^23^. The mixed conditions were found in: Anaya *et al.* ^16^, Mehta *et al.* ^20^, Mancera-Páeza *et al.* ^24^. Of the 20 subjects, 11 (55%) were women, 7 (35%) were in the 20-30 age group (23 mode and 26 median) and 8 (40%) had previous immunization against arbovirus. Regarding treatment, 17/20 (85%) received corticosteroid therapy, and 8/20 (40%) had a score greater than 3 on the modified Rankin scale (mRS). The mode and median of the prodromes were 7 days.

ATM group:

The distribution between the sexes was equivalent, with 7/14 subjects in each. The mode and median age were 23 years, and the mode and median of prodrome days were 7 days.

Regarding the clinical picture, of the 14 subjects, 13 (92%) had altered sensitivity, 11 (78%) had altered reflexes and 10 (71%) had lower limb paresis or paralysis. Vegetative symptoms such as cardiac arrhythmia were present in 2/14 (14%), and blood pressure lability in 1/14 (7%). Convulsive crisis and syncope were described in a case with a previous diagnosis of Systemic Lupus Erythematosus (SLE) (article 3). In 1/14 (7%) there was coinfection, with Zika, dengue and chikungunya viruses simultaneously. Autoantibodies were found in 2/14 (14%). In one case, autoantibodies against oligodendrocyte myelin glycoprotein (Anti-MOG) were found and in the other, antiganglioside autoantibodies GM1, GD1a and GD1b.

Thirteen (92%) of the 14 subjects received corticosteroid therapy and 6/14 (42%) had mRS less than or equal to 3. In 7/14 (50%) of the subjects there was no data on sequelae.

Mixed group:

Six individuals had broader involvement, 3 with ATM-associated encephalitis and 3 with ATM-associated radiculitis. Among the 3 patients with encephalitis, one had Guilin-Barré syndrome and the other had subclinical meningitis. The mode of the prodromes was 1 day and the median was 2 days. Of the 6, 4 were women, and 3 were over 50 years old. The age mode was 26 years old, and the median was 41 years old. Coinfection with other arboviruses occurred in 4/6 subjects, being 3 Chikungunya and 1 dengue.

**Figure 2:**
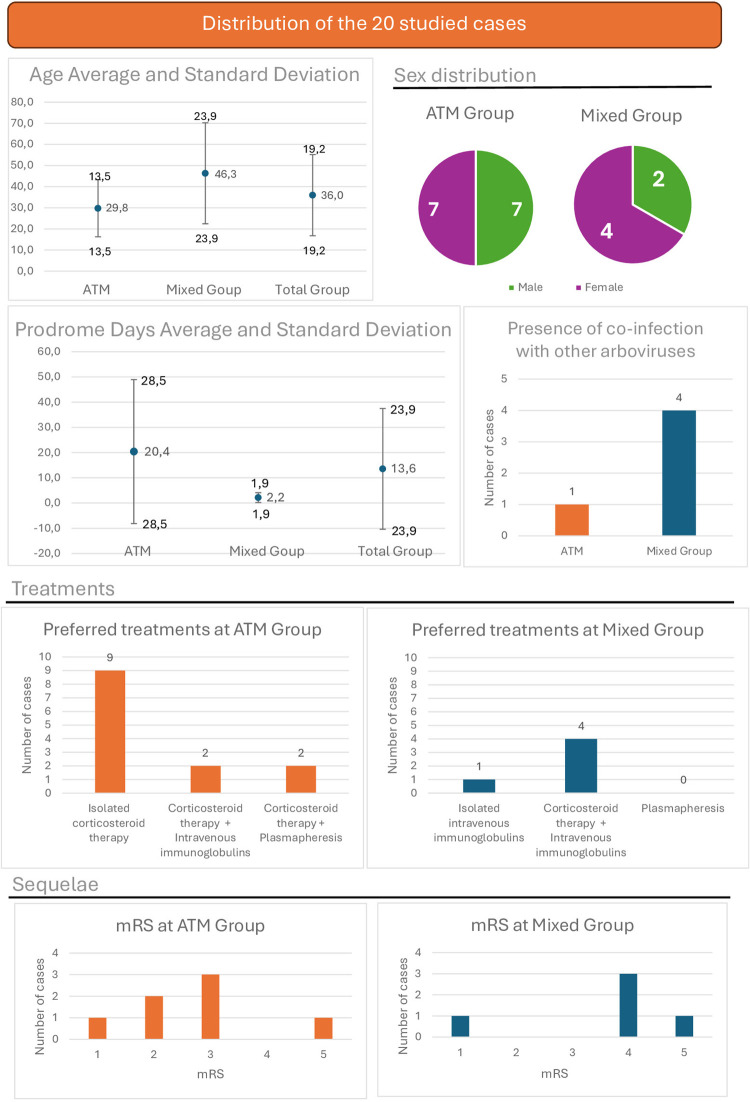
Distribution of the 20 studied cases in terms of their ages, sex, prodrome duration, presence of coinfection, preferred treatments and mRS scores.

Regarding the clinical picture, the alteration of the reflexes was present in all; 5/6 presented altered sensitivity, and 4/6 urinary retention. The treatment was described for 5 subjects. Of these, one received immunoglobulin alone, and 4, associated with corticosteroid therapy. Regarding the degree of sequelae, 5/6 subjects presented mRS 4 or higher. The results are summarized in Figure 2.

## DISCUSSION

The objective of this study was to survey the clinical/diagnostic characteristics, treatment and evolution of cases of acute transverse myelitis associated with ZIKV infection. Thus, 9 articles described these characteristics, totaling 20 individuals.

As Brazil was the epicenter of the epidemic, Brazilian publications predominated, followed by Latin America and the French Caribbean. Most articles were published between 2017 and 2018, the height of the epidemic. ATM is a rare post-infectious inflammatory disease. Thus, the low number of publications may reflect this rarity, since there were no publications with a large number of cases, even by research groups formed in the epidemic.

The age distribution of ATM is bimodal, with peaks between 10 and 19 years, and between 30 and 39 years^10^. Among the published cases, there was a predominance of the 20-30 age group. Among the six mixed, 3 were over 55 years old, contrasting with 1 case in the group of isolated ATMs. All 3 cases evolved with 4-5 mRS. The low number of published cases makes inference difficult, however, this is a population with greater propensity to morbidities and lower immunological vitality^25^. Thus, the clinician must be vigilant for more diffuse and more severe conditions in this age group.

The prodrome time was considerably shorter in the mixed group (2 days mean, compared to 7 for isolated ATM.), suggesting that more severe cases manifest earlier, or that the shorter the prodrome time, the greater the possibility of multiple involvement of the central and peripheral nervous system.

In 12 subjects it was possible to determine the mRS. Of these, only 3 had mRS ≤ 2, two with MTA alone. The 4 aged over 55 years had moderate to severe sequelae by the mRS score. It is, therefore, a serious disease with a high risk of sequelae in the medium term. Among the 7 subjects in which it was possible to calculate mRS in the MTA group alone, 6 had mRS up to 3, while 5/6 patients in the mixed group had mRS of 4 or higher, suggesting that mixed conditions are associated with more debilitating sequelae. However, only in seven subjects the follow-up time was described, which varied from 1 to 3 months. The recovery period may last for years post-event^10^, and the mRS score may change. In the individual with SLE, a risk factor for unfavorable evolution, there was no description of the evolution.

The literature is controversial regarding the predominance of sex. Beh *et al*.^8^ describe predominance in women, whereas Greenberg *et al*.^10^ do not report predominance. In this review, no difference was found, with 11 females and 9 males. On the other hand, 4/6 subjects in the mixed group were female, whereas in the isolated ATM group the proportion was exact 7:7. When evaluating worse evolution (mRS ≥ 3), 5/8 were women. Although there was no predominance of sex, this review suggests that women were more likely to have diffuse (mixed) conditions, experienced worse outcomes, and were also over 55 years old. Autoimmune diseases are more frequent in females^26^, but this may be reflected in ATM severity.

ATM is a spinal cord syndrome. Sensitivity changes must be present. In one subject (article 3) there was no description of the presence or absence of the alteration. In 3/14 subjects there were signs of dysautonomia, such as syncope, postural lability and arrhythmia (articles 1 and 3), which are rarely seen in idiopathic ATMs^16,24^. One of these patients, who developed syncope and seizures, had a previous diagnosis of SLE (article 3), which may have contributed to or triggered the development of these symptoms^27^.

In the mixed group, there was one subject who presented no changes in sensitivity (article 5 – F 80 years old). In this article, the authors describe that there was no change in light touch, vibration, temperature and proprioception that were perceptible on physical examination, in addition to no apparent sensory level, however, the reason for this to have occurred is unknown.

Metha *et al*.^20^ hypothesized that the existence of previous immunity against the dengue virus could be a risk factor for developing neurological complications after ZIKV infection, since they found positive IgG for dengue in some patients who progressed with greater severity. The study by Dejnirattisai *et al*.^28^ demonstrated that antibodies against dengue were able to stimulate the Zika virus in vitro. Bardina *et al*.^29^ also reported higher morbidity and mortality in mice infected with Zika after injection of plasma containing antibodies against DENV and West-Nile virus. Although 8/20 (40%) of the subjects in this review had a history of previous arbovirus infection, only these subjects were serologically tested or reviewed their medical records in search of these infections (articles 1 and 9).

Metha *et al*.^20^ believe that simultaneous coinfections between arboviruses would lead to more severe neurological cases. As the clinical picture of arboviruses is superimposable, positive serology for more than one arbovirus would not define whether the disease is being caused by one of them or by both simultaneously. In this review, the mixed group had 4 individuals with more than one arbovirus, while the ATM group had only 1. In view of the possibility of worse morbidity and mortality and overlapping infections, IgG measurement against dengue and chikungunya could be performed in all cases with neurological manifestations after a ZIKV infection.

There is cross-reactivity between flaviviruses, making serological diagnosis difficult, and may even point out coinfections erroneously. Tests using PRNT (plaque reduction neutralization testing) are more accurate, but cross-reactivity is not completely eliminated. However, they have the disadvantage of being expensive and requiring specialized laboratories^20,23,30^. In none of the 20 subjects in the review this method was performed, most likely due to these disadvantages.

The pathophysiology of post-zika virus infection myelitis is not yet fully understood. Myelitis can be caused by direct spinal cord microbial infection, immune-mediated spinal cord injury, or delayed systemic response leading to neural injury^11,31^. The most accepted hypothesis is that myelitis after Zika virus infection has an autoimmune etiology, due to immune-mediated damage after direct spinal cord infection (Zika virus is neurotoxic^16,32^); or by a systemic immune reaction caused by infection at other sites^10,16^. Fernandes *et al*.^33^ observed that rats developed myelitis on the 12th day after inoculation with a Zika virus strain isolated in Brazil during the 2015 epidemic, but it was not possible to conclude whether due to direct spinal cord infection.

In this review, there were two positive cases for autoantibodies (articles 5 and 6), one with GM1, GD1a and GD1b anti-gangliosides, and another with anti-MOG, despite the fact that most authors performed tests. There was one case of autoimmune disease—SLE—diagnosed 15 years ago, in remission.

Anti-GM1 and anti-GD1a antibodies are associated with the axonal motor variant of Guillain Barré Syndrome (AMAN), whereas anti-GD1b with ataxic sensory neuropathy^12^. According to Metha *et al*.^20^, the meaning of this association is yet to be clarified. Anti-MOG is an antibody against myelin oligodendrocyte glycoproteins, a component found in the central nervous system that is associated with demyelinating autoimmune diseases such as Neuromyelitis Optica and Multiple Sclerosis. There is a hypothesis of a cross-immunological response between the ZIKV and these glycoproteins^21^. Neri *et al*.^21^ report that the number of MOG-mediated demyelinations that present a previous infectious condition is increasing, suggesting a para-infectious etiology.

In this review, one subject had a previous autoimmune disease—SLE (article 3). The authors did not describe the evolution. This information would enable comparisons between that case with the others in this review. The panel of antibodies tested was negative (Anti-DNA, anti-Cardiolipin IgM, Beta-2-Glycoprotein and Anti-Aquaporin), suggesting ZIKV infection as a cause of ATM. However, it presented a more restricted picture, with isolated ATM, leading to the assumption that it could be associated with immunosuppressive treatment of SLE^18^.

The literature brings the possibility of treatment with corticosteroids, intravenous immunoglobulins (IVIG) or plasmapheresis^10^. The first two are the most used. Plasmapheresis was chosen for two subjects (articles 7 and 8), in one case due to the therapeutic failure of corticosteroid therapy, while in the other, no reference was made to the medical rationale for the choice. In the group of mixed diseases, IVIG alone or in combination with corticosteroids were used more often, whereas isolated corticosteroid therapy was preferred in isolated ATMs.

As previously described, the worst sequelae occurred in the mixed condition group. Only one case presented mRS 5 in the group of isolated ATMs (article 2). The case characteristics were atypical in relation to others with a worse prognosis: young (23 years old), sex (male) and isolated ATM group. However, prednisone 0.5 mg/kg/day was used for treatment (article 2). The recommended treatment regimens are methylprednisolone 30 mg/kg/day, or dexamethasone 200 mg daily, for 3 to 5 days, followed by plasmapheresis or IVIG in case of therapeutic failure^10^. In this patient, none of these regimens were used. Therefore, it can be questioned whether the worse evolution was due to treatment differences.

The limitations of this review are due to the different diagnostic methodologies used by different authors, and the small number of cases, which can be sources of bias. In addition, the mRS was roughly assigned by the author, based on the descriptions provided in the articles, and thus may be imprecise.

## CONCLUSIONS

This review suggests that post-Zika virus infection MTA occurs either alone or accompanied by other neurological manifestations, especially encephalitis and radiculitis. Short prodrome time and age over 55 years suggest attention to mixed cases. Advanced age, female gender, and broader nervous system involvement were associated with worse sequelae at follow-up. Attention should also be paid to coinfection with other arboviruses, as it may predispose to greater severity of the conditions and the occurrence of mixed diseases.
